# Landscape‐scale genetic differentiation of a mycangial fungus associated with the ambrosia beetle, *Xylosandrus germanus* (Blandford) (Curculionidae:Scolytinae) in Japan

**DOI:** 10.1002/ece3.3437

**Published:** 2017-10-03

**Authors:** Masaaki Ito, Hisashi Kajimura

**Affiliations:** ^1^ Graduate School of Bioagricultural Sciences Nagoya University Nagoya Japan

**Keywords:** *Ambrosiella hartigii*, coevolution, mycangium, phylogeography, *Xylosandrus germanus*

## Abstract

In this study, we examined the genetic structures of the ambrosia fungus isolated from mycangia of the scolytine beetle, *Xylosandrus germanus* to understand their co‐evolutionary relationships. We analyzed datasets of three ambrosia fungus loci (18S rDNA, 28S rDNA, and the β‐tubulin gene) and a *X. germanus* locus dataset (cytochrome c oxidase subunit 1 (*COI*) mitochondrial DNA). The ambrosia fungi were separated into three cultural morphptypes, and their haplotypes were distinguished by phylogenetic analysis on the basis of the three loci. The *COI* phylogenetic analysis revealed three distinct genetic lineages (clades A, B, and C) within *X. germanus*, each of which corresponded to specific ambrosia fungus cultural morphptypes. The fungal symbiont phylogeny was not concordant with that of the beetle. Our results suggest that *X. germanus* may be unable to exchange its mycangial fungi, but extraordinary horizontal transmission of symbiotic fungi between the beetle's lineages occurred at least once during the evolutionary history of this symbiosis.

## 1 INTRODUCTION

The associations among insects and fungi are highly diverse (Vega & Blackwell, 2005). Fungivorous insects include more than 150 families in 33 orders and reach 10% in the species of Insecta (Hammond & Lawrence, 1989). Insects also show a vast array of symbiotic relationships with a wide diversity of fungi. These relationships may confer a variety of benefits to the host insects, such as direct or indirect nutrition, the ability to counter the defenses of plant or animal hosts, protection from natural enemies, improved development and reproduction, and communication (Klepzig, Adams, Handelsman, & Raffa, 2009). Some insects, particularly fungus‐growing ants, fungus‐growing termites, and ambrosia beetles, rely on the cultivation of fungi for food, and these cultivated fungi are a nutrition source for larvae and adults (Mueller, Gerardo, Aanen, Six, & Schultz, 2005).

Ambrosia beetles have a close association with symbiotic fungi and carry the inoculum of symbiotic fungi in special structures (mycangia; Beaver, 1989). Ambrosia beetles bore into the xylem of woody plants and feed on fungi that they culture on the walls of the tunnels in the wood. The fungi may supply sterols and B‐group vitamins important for beetle development (Kok, Norris, & Chu, 1970). Approximately 3,400 species of ambrosia beetles are found in 10 tribes of two subfamilies of Curculionidae, Scolytinae, and Platypodinae (Farrell et al., 2001). The xylomycetophagous habit is considered to have evolved at least seven times in Scolytinae (Farrell et al., 2001). Different beetle species in Scolytinae have different symbiotic associations with their own specific fungi (Kajimura & Hijii, 1994). Symbiotic fungi associated with scolytine beetles are polyphyletic and comprise two primary ophiostomatoid clades that include *Ceratocystis* and *Ophiostoma* (Farrell et al., 2001). The two genera are not closely related, and their ancestors may have diverged more than 170 million years ago (Farrell et al., 2001). Thus, ambrosia beetles strongly depend on polyphyletic fungal groups.

*Xylosandrus germanus* (Blandford) is a common ambrosia beetle in the subfamily Scolytinae. This species was originally distributed in eastern and southeastern Asia (Nobuchi, 1981; Wood & Bright, 1993) but has now invaded central Europe, North America, and Hawaii (Bouget & Noblecourt, 2005; Cognato & Rubinoff, 2008; Grégoire, Piel, Proft, & Gilbert, 2001; Henin & Versteirt, 2004; Lakatos & Kajimura, 2007; López, Iturrondobeitia, & Goldarazena, 2007; Rabaglia, 2003; Rabaglia, Dole, & Cognato, 2006; Wanat & Mokrzycki, 2005; Weber & McPherson, 1983a; Wood & Bright, 1993). The beetle has at least 220–264 host species worldwide (Weber & McPherson, 1983b) and 146 species in Japan (Nobuchi, 1981). *X. germanus* has a much wider range of hosts than that other ambrosia beetles, and thus, it can colonize a range of forest types (Henin & Versteirt, 2004). It has become a serious forest pest in many countries, regardless of whether it is native or exotic in those countries (Grégoire et al., 2001; Kaneko, Tamaki, & Takagi, 1965; Nobuchi, 1981 Weber & McPherson, 1983a, 1984).

*Xylosandrus germanus* biology was well‐documented by Kaneko and Takagi (1966). Only females disperse; bore into stems, twigs, and roots of susceptible woody plants; excavate a gallery system in the wood or pith; introduce symbiotic fungi; and produce a brood. The female parent remains with her brood until they are mature. Adults and larvae feed on the ambrosia fungus introduced by the female parent. The sex ratio is strongly female‐biased; males are rare, reduced in size, and flightless. Young females mate with their brothers (inbreeding) before emerging to attack a new host. Males are haploid, and females are diploid (Takagi & Kaneko, 1966). *Ambrosiella hartigii* Batra, the symbiotic fungi of *X. germanus*, is common to Japan, China, the United States, and Germany (Batra, 1967; Weber & McPherson, 1984; Yang, Ye, & Zhang, 2008). *A. hartigii* has been isolated from adult female mycangia, except for the callow adult (Kaneko & Takagi, 1966; Yang et al., 2008). *Ambrosiella hartigii* was isolated from *X. germanus* and *Anisandrus dispar* (Fabricius) mycangia (Batra, 1967). Mayers et al. (2015) showed fungal symbiont isolated from *A. dispar* and *X. germanus* was *A. hartigii* and *Ambrosiella grosmanniae* Mayers, McNew & Harr., respectively, using molecular methods and morphology.

A phylogenetic analysis based on cytochrome oxidase I (*COI*) mitochondrial DNA (mtDNA) revealed three distinct lineages (clades A, B, and C) within *X. germanus* in Japan (Ito, Kajimura, Hamaguchi, Araya, & Lakatos, 2008). The rates of substitutions per site between the three lineages are 12.4%–15.0%, which are similar to those calculated as differences among scolytine beetle species in the genera *Ips*,* Tomicus*, and *Dendroctonus* (Cai, Cheng, Xu, Duan, & Kirkendall, 2008; Cognato & Sperling, 2000; Duan, Kerdelhué, & Lieutier, 2004; Lakatos, Grodzki, Zhang, & Stauffer, 2007; Maroja, Bogdanowicz, Wallin, Raffa, & Harrison, 2007). Thus, these different *X. germanus* lineages may have genetically different fungi in their mycangia.

In this study, we investigated the genetic structure of an ambrosia fungus isolated from *X. germanus* mycangia and adult females used for fungal isolation in order to elucidate the differentiated fungal and beetle lineage patterns. We also discuss evolutionary events that may have influenced the diversification of their mutualistic system.

## 2 MATERIALS AND METHODS

### 2.1 Insect collection and fungi isolation

We collected *X. germanus* samples from 14 sites in Japan (Table 1). To capture live adult females, in 2007, we set up Nagoya University (Meidai) traps (Ito & Kajimura, 2006) baited with 99.5% ethanol at all sites and used 1–31 mature females from each site (Table 1). We also trapped adult females of *Xylosandrus brevis* (Eichhoff) in Aichi Prefecture (AIT) and *Scolytoplatypus mikado* (Blandford) in AIT and Wakayama Prefecture (WKT). Two species of *Xylosandrus* beetles and *S. mikado* were identified according to Nobuchi (1981, 1980), respectively.

**Table 1:** Description of *Xylosandrus germanus* samples used for isolations of mycangial fungi

Sampling site	Acronym	Regions	Areas	Latitude (N)	Longitude (E)	Altitude (m)	No. of samples^a^
Furano, Hokkaido Pref.	HKF	Northern Japan	Hokkaido	43° 10′	142° 20′	500–700	29 (25)
Sapporo, Hokkaido Pref.	HKS	Northern Japan	Hokkaido	43° 00′	141° 23′	70	31 (11)
Iwate‐gun, Iwate Pref.	IWI	Northern Japan	Tohoku	39° 53′	141° 10′	190	7 (7)
Tsuruoka, Yamagata Pref.	YMT	Northern Japan	Tohoku	38° 39′	139° 49′	210	23 (11)
Chichibu, Saitama Pref.	SIC	Eastern Japan	Kanto	35° 55′	138° 50′	500	21 (10)
Shimominochi‐gun, Nagano Pref.	NGM	Central Japan	Chubu	37° 00′	138° 32′	670	2 (2)
Chi'isagata‐gun, Nagano Pref.	NGC	Central Japan	Chubu	36° 32′	138° 21′	1,300	22 (10)
Shiojiri, Nagano Pref.	NGS	Central Japan	Chubu	36° 08′	138° 00′	790	4 (4)
Nakashinkawa‐gun, Toyama Pref.	TYN	Central Japan	Hokuriku	36° 36′	137° 20′	260	23 (10)
Toyota, Aichi Pref.	AIT	Central Japan	Tokai	35° 12′	137°34′	930–1,070	25 (10)
Nantan, Kyoto Pref.	KTN	Western Japan	Kinki	35° 16′	135° 30′	250	23 (10)
Tanabe, Wakayama Pref.	WKT	Western Japan	Kinki, Kii peninsula	33° 42′	135° 33′	500	1 (1)
Miyoshi, Hiroshima Pref.	HRM	Western Japan	Chuugoku, Sanyo	34° 47′	132° 51′	200	27 (11)
Hata‐gun, Kouchi Pref.	KUH	Western Japan	Shikoku	33° 12′	133° 02′	1,336	22 (10)

a The numbers in bracket are the number of individuals used in the DNA analysis of both *X. germanus* and its symbiotic fungi.

We isolated fungal conidia from mycangia of *X. germanus*,* X. brevis*, and *S. mikado* living adult females. All collected beetles were preserved at −20°C in 99.5% ethanol after fungal isolation. Isolates from mycangia were directly placed on potato dextrose agar (PDA) plates in 90‐mm sterile Petri dishes and incubated at 20°C for 5 days in the dark. The isolates were grouped by cultural characteristics and identified at the generic level using the ambrosia fungi keys of Batra (1967).

### 2.2 DNA extraction, polymerase chain reaction (PCR), and DNA sequencing

Total DNA was extracted using the methods of Walsh, Metzger, and Higuchi (1991) and Suzuki, Taketani, Kusumoto, and Kashiwagi (2006), with some modifications. A small amount of mycelium was scraped from the surface of cultures grown on PDA, and all muscle tissue from the abdomen of each adult female was sampled to extract DNA. The mycelium and muscle tissue were macerated in 200 μl of Chelex 100 sodium (0.26 g/5 ml, Bio‐Rad Laboratories, Hercules, CA, USA) and 4 μl of Proteinase K (600 mAU/ml, Qiagen, Valencia, CA, USA). The samples were incubated at 56°C for at least 10 hr. After incubation, the samples were vortexed for 10 s and then heated at 99°C for 3 min to inactivate the proteinase. The solutions were vortexed again for 10 s and centrifuged at 15,027 *g* for 2 min. The supernatant was adjusted to a standard mixture density (1 ng/μl) by adding Tris EDTA (pH 8.0) and used for PCR analysis.

PCR amplification for the symbiotic fungi was performed using the primer pairs NS1 (5′‐GTA GTC ATA TGC TTG TCT C‐3′; White, Bruns, Lee, & Taylor, 1990) and NS4 (5′‐CTT CCG TCA ATT CCT TTA AG‐3′; White et al., 1990), NL1 (5′‐GCA TAT CAA TAA GCG GAG GAA AAG‐3′; O'Donnell, 1993) and NL4 (5′‐GGT CCG TGT TTC AAG ACG G‐3′; O'Donnell, 1993), and Bt2a (5′‐GGT AAC CAA ATC GGT GCT GCT TTC‐3′; Glass & Donaldson, 1995) and Bt2b (5′‐ACC CTC AGT GTA GTG ACC CTT GGC‐3′; Glass & Donaldson, 1995) to amplify a portion of the small subunit (18S) rDNA, large subunit (28S) rDNA, and partial β‐tubulin genes, respectively. PCR amplification for the insects was performed using the primer pairs C1‐J‐2183 (5′‐CAA CAT TTA TTT TGA TTT TTT GG‐3′; Simon et al., 1994) and TL‐2‐N‐3014‐ANT (5′‐TGA AGT TTA AGT TCA ATG CAC‐3′; Ito et al., 2008) to amplify a portion of the *COI* mtDNA gene. For the PCR analysis, we mixed 1 μl of extracted DNA, 2 μl of each primer (5 pmol/μl), 0.8 μl of dNTPs (Takara, Otsu City, Shiga, Japan), 1 μl of 10 ×  PCR buffer (Takara), 0.1 μl of Taq DNA polymerase (5 units/μl, Takara), and 3 μl of distilled water in a total volume of 10 μl. The PCR conditions for NS1/NS4 were as follows: one cycle of denaturation at 94°C for 2 min; followed by 40 cycles of denaturation at 94°C for 1 min, annealing at 56°C for 1 min, and extension at 72°C for 2 min; and one final cycle of extension at 72°C for 2 min. The PCR conditions for NL1/NL4 and Bt2a/Bt2b were as follows: one cycle of denaturation at 94°C for 2 min, followed by 40 cycles of denaturation at 94°C for 2 min and annealing at 54°C for 1 min, and one final cycle of extension at 72°C for 10 min. The PCR conditions for C1‐J‐2183/TL‐2‐N‐3014‐ANT were as follows: one cycle of denaturation at 94°C for 1 min; followed by 40 cycles of denaturation at 94°C for 1 min, annealing at 48°C for 1 min, and extension at 72°C for 2 min; and one final cycle of extension at 72°C for 2 min. The samples were refrigerated at 4°C until the reaction tubes were removed from the PCR machine.

The PCR products were purified using a QIAquick PCR purification kit (Qiagen). Direct sequencing was performed using the ABI Big Dye Terminator v3.1 Cycle Sequencing Kit (Applied Biosystems, Foster City, CA, USA) with the same primer sets as used for the PCR reactions. Sequencing was performed using an ABI PRISM‐3100 Genetic Analyzer (Applied Biosystems).

### 2.3 Data analysis

Sequences were aligned using the BioEdit v.7.0.2 software (Hall, 1999). BLAST searches were performed with sequences of each isolate in the NCBI GenBank database (http://www.ncbi.nlm.nih.gov), and published sequences of relevant and related species were incorporated into the datasets (Tables 2 and 3). Calculations for the G‐C composition were performed using the MEGA 4 software (Tamura, Dudley, Nei, & Kumar, 2007). For the phylogenetic analysis, we chose maximum‐parsimony (MP) method (Nei & Kumar, 2000), using the MEGA 4 software. The MP analysis also used 1,000 bootstrap replications. A phylogenetic analysis was also performed for the three loci (18S, 28S, and β‐tubulin) using MP methods. Concordance among the three different gene datasets was evaluated by the incongruence length difference (ILD) test (Farris, Källersjö, Kluge, & Bult, 1995) implemented with PAUP*4.0b10 (Swofford, 2003), using 1,000 replicates.

**Table 2:** List of ambrosia fungi used in this study, including their sequences obtained from GenBank

Species	Source	GenBank accession no.			References
		18S rDNA	28S rDNA	β‐tubuline gene	
*Ambrosiella batrae*	C3130	KR673881	—	—	Mayers et al. (2015)
*Ambrosiella beaveri*	CBS 121751	—	EU825650	EU825656	Six, Stone, de Beer, and Woolfolk (2009)
	CMW26179	—	KM495315	—	De Beer et al. (unpublished)
	PL5329	—	KT803728	—	Bateman et al. (unpublished)
	C2749	KR673882	KF646765	—	Harrington, McNew, Mayers, Fraedich, and Reed (2014)
*Ambrosiella grosmanniae*	C3151	KR673884	—	—	Mayers et al. (2015)
	1002HHS1	—	LC175288	—	Lin et al. (unpublished)
*Ambrosiella nakashimae*	C3445	KR673883	—	—	Mayers et al. (2015)
	0414XX7	—	LC175285	—	Lin et al. (unpublished)
*Ambrosiella hartigii*	TUB F4276	AY858656	—	—	Gebhardt, Weiss, and Oberwinkler (2005)
	CBS 403.82	—	AF275506	—	Paulin‐Mahady, Harrington, and McNew (2002)
	CBS 404.82	EU984256	EU984288	EU977463	Alamouti, Tsuib, and Breuil (2009)
	CMW20920	—	—	EU825654	Six et al. (2009)
	CMW25525	—	KM495317	—	De Beer et al. (unpublished)
	C1573	KR673885	—	—	Mayers et al. (2015)
*Ambrosiella roeperi*	C2448	KR673886	KF646767	—	Mayers et al. (2015)
	B239U1	—	LC175297	—	Lin et al. (unpublished)
*Ambrosiella xylebori*	CBS 110.61	AY858659	—	—	Gebhardt et al. (2005)
	CBS 110.61	—	EU984294	EU977469	Alamouti et al. (2009)
	AFTOL‐ID 1285	DQ471031	DQ470979	—	Spatafora et al. (2006)
	C3051	KR673887	—	—	Mayers et al. (2015)
	Hulcr5114	KU961668	KU961669	—	Bateman, Sigut, Skelton, Smith, and Hulcr (2016)
*Ceratocystis adiposa*	CMW2573	—	KM495320	—	De Beer et al. (unpublished)
	CCFC212707	—	AY283562	—	Seifert, Louis‐Seize, and Sampson (2003)
	CBS600.74	EU984263	EU984304	EU977479	Alamouti et al. (2009)
	VPCI 2818/12	—	KF060720	—	Agarwal et al. (2014)
*Ceratocystis fagacearum*	C999	KR673891	—	—	Mayers et al. (2015)
*Ceratocystis major*	C927	KR673892	—	—	Mayers et al. (2015)
*Ceratocystis fimbriata*	CMW3189	—	KM495350		De Beer et al. (unpublished)
	C1099	KR673893	—	—	Mayers et al. (2015)
	CBS 146.53	U43777	—	—	Issakainen, Jalava, Eerola, and Campbell (1997)
*Ceratocystis norvegica*	WIN(M)87	DQ318209	—	—	Reid, Iranpour, Rudski, Loewen, and Hausner (2010)
	C3124	KR673894	—	—	Mayers et al. (2015)
*Meredithiella norrisii*	C3152	KR673888	—	—	Mayers et al. (2015)
*Phialophoropsis ferruginea*	CBS 408.68	—	—	EU825653	Six et al. (2009)
	CBS 408.68	—	AF275505	—	Paulin‐Mahady et al. (2002)
	CBS 378.68	EU984254	EU984285	EU977461	Alamouti et al. (2009)
	JB13	EU984255	EU984286	EU977462	Alamouti et al. (2009)
	CBS 460.82	—	EU825651	EU825652	Six et al. (2009)
	M243	KR673889	—	—	Mayers et al. (2015)
*Phialophoropsis* sp.	CBS460.82	KR673890			Mayers et al. (2015)

**Table 3:** List of ambrosia beetles used in this study, including their sequences obtained from GenBank

Species	GenBank accession no.	References
	*COI* mtDNA	
*Xylosandrus germanus*	AB373682	Ito et al. (2008)
	AB373683	Ito et al. (2008)
	AB373684	Ito et al. (2008)
	AB373703	Ito et al. (2008)
	AB373704	Ito et al. (2008)
	AB373711	Ito et al. (2008)
	EF433438	Lakatos & Kajimura (2007)
	EF433439	Lakatos & Kajimura (2007)
*Xylosandrus brevis*	AB476316	Ito & Kajimura (2009a)
*Xylosandrus crassiusculus*	AB462579	Ito & Kajimura (2009b)

## 3 RESULTS

### 3.1 Morphological characters of symbiotic fungi isolated from X. germanus mycangia

**Figure 1:**
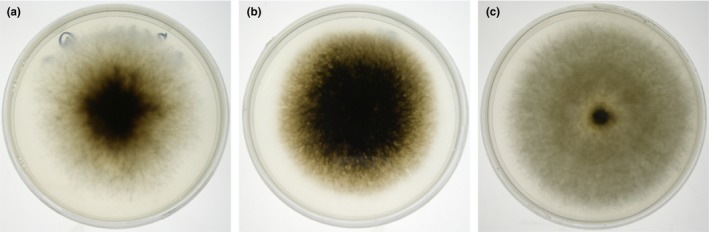
Colonies of symbiotic fungi isolated from *Xylosandrus germanus* mycangia. (a) Type I. (b) Type II. (c) Type III. Fungal cultures were held at 20°C for 5 days on potato dextrose agar in the dark

Based on the color and growth pattern of colonies (mycelia tuft), isolates obtained from *X. germanus* mycangia were separated into three cultural types (Types I, II, and III) (Figure 1). Five days after inoculation, colony characteristics of Types I and II were similar to those of *A. hartigii* and *A. grosmanniae* shown in Batra (1967) and Mayers et al. (2015), respectively, but aerial mycelia were observed only in Type I colonies. Five day old Type III colonies were tinged with white on agar media. Type III colonies did not have cottony aerial mycelia as the Type I colonies.

**Figure 2:**
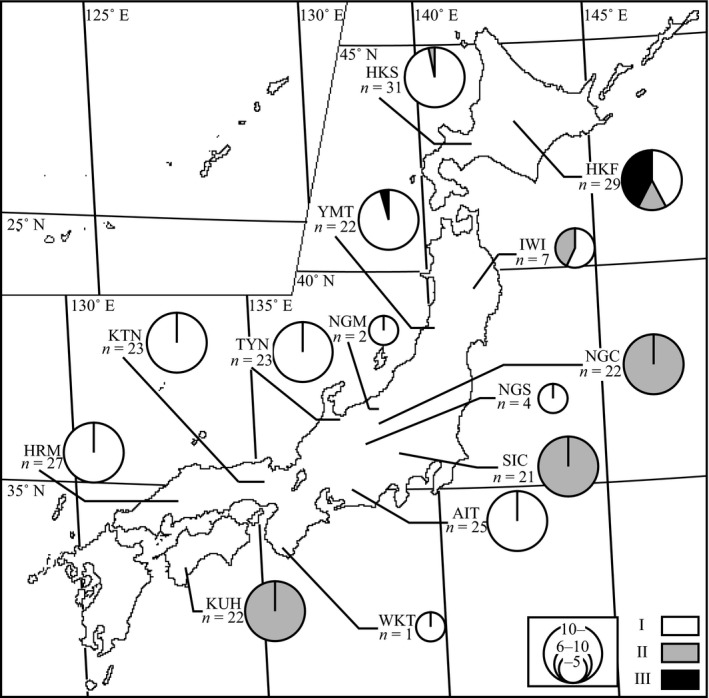
Distribution of symbiotic fungi cultural types across 14 Japanese *Xylosandrus germanus* populations. Pie charts represent frequencies of Types I–III (Figure 1) in each population. The size of the circle is proportional to the sample size. Population names are defined in Table 1

Type I was found in all 11 populations (HKF, HKS, IWI, YMT, NGM, NGS, TYN, AIT, KTN, WKT, and HRM), except for SIC, NGC, and KUH; Type II was found in six northern, eastern, central, and western populations (HKF, HKS, IWI, SIC, NGC, and KUH); and Type III was only found in two northern populations (HKF and YMT; Figure 2). Type I fungi were distributed throughout Japan, but the other two types were located in Japan.

### 3.2 DNA sequencing and phylogenetic analyses

**Figure 3:**
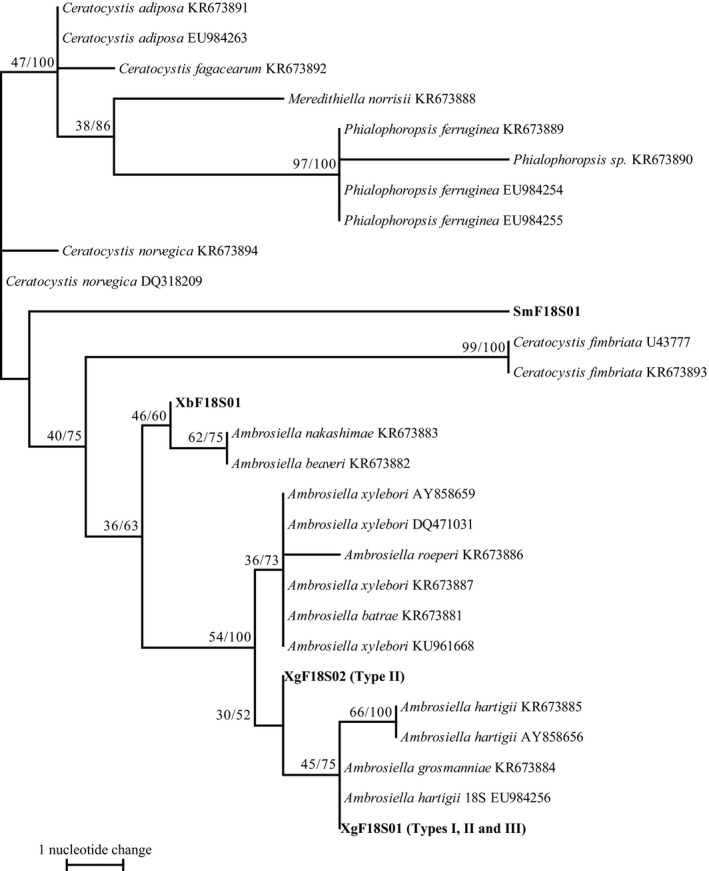
Phylogram obtained from 997 bp of the 18S rDNA of symbiotic fungi isolated from mycangia of *Xylosandrus germanus* and related fungus species. One of 228 maximum‐parsimony (MP) trees. CI = 0.741, RI = 0.920, length = 40 steps. Bootstrap values (left) and branch support values (right) (>50%) are given above the branches. Bold letters indicates sequences obtained in this study. Cultural types (Type I‐III) defined in Figure 1 are shown in bracket after haplotype codes (XgF18S01‐02). XbF and SmF represent symbiotic fungi isolated from mycangia of *Xylosandrus brevis* and *Scolytoplatypus mikado*, respectively

The amplicons obtained from the 18S regions of ambrosia fungi sequenced in this study were 997 bp in length. These fragments had 45.5% G/C content. Two haplotypes were defined from 132 isolates. Haplotype XgF18S01 was detected in all cultural types (Figure 3). Haplotype XgF18S02 was detected only in Type II. The haplotypes of ambrosia fungi isolated from *X. germanus* mycangia were clustered as a monophyletic group with those of *A. grosmanniae* (KR673884), *A. hartigii* (AY858656, EU984256, and KR673885), *Ambrosiella xylebori* Brader ex Arx & Hennebert (AY858659, DQ471031, KR673887, and KU961668), *Ambrosiella roeperi* Harr. & McNew (KR673886), and *Ambrosiella batrae* Mayers, McNew & Harr. (KR673881).

**Figure 4:**
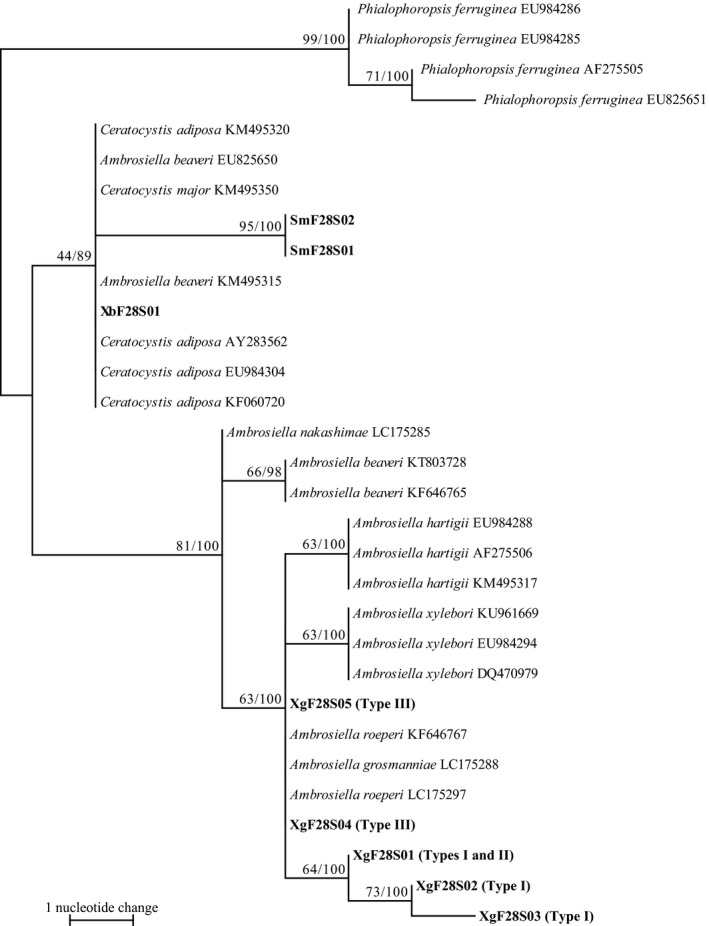
Phylogram obtained from 607 bp of the 28S rDNA of symbiotic fungi isolated from mycangia of *Xylosandrus germanus* and related fungus species. One of 376 maximum ‐parsimony (MP) trees. CI = 0.900, RI = 0.979, length = 22 steps. Bootstrap values (left) and branch support values (right) (>50%) are given above the branches. Bold letters indicate sequences obtained in this study. Cultural types (Type I‐III) defined in Figure 1 are shown in bracket after haplotype codes (XgF28S01‐05). XbF and SmF represent symbiotic fungi isolated from mycangia of *Xylosandrus brevis* and *Scolytoplatypus mikado*, respectively

Sequencing of the 28S rDNA from *A. hartigii* aligned 607 bp. These fragments had 47.6% G/C content. Five haplotypes were defined from 132 isolates. Haplotypes XgF28S01, XgF28S02, and XgF28S03 were detected in two culture types (Types I and II) (Figure 4). Haplotypes XgF28S04 and XgF28S05 were detected only in Type III. The *X. germanus* fungi haplotypes were clustered as a monophyletic group with those of *A. grosmanniae* (LC175288), *A. roeperi* (KU961669 and LC175297), *A. hartigii* (AF275506, EU984288, and KM495317), and *A. xylebori* (DQ470979, EU984294, and KU961669). Within this clade, three haplotypes of *X. germanus* fungi, XgF28S01–03, and *A. hartigii* and *A. xylebori* were clustered with high bootstrap value of 64, 63, and 63, respectively. Within XgF28S01–03 clade, two haplotypes, XgF28S02 and 03, were clustered with high bootstrap value of 73. Five haplotypes of *X. germanus* fungi were not clustered as a monophyletic group.

**Figure 5:**
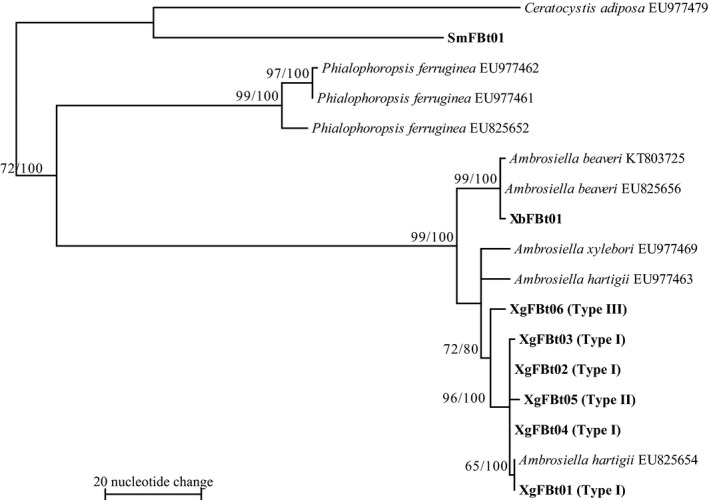
Phylogram obtained from approximately 440 bp of the β‐tubulin gene from symbiotic fungi isolated from mycangia of *Xylosandrus germanus* and related fungus species. One of 56 maximum‐parsimony (MP) trees. CI = 0.840, RI = 0.898, length = 358 steps. Bootstrap values (left) and branch support values (right) (>50%) are given above the branches. Bold letters indicate sequences obtained in this study. Cultural types (Type I–III) defined in Figure 1 are shown in bracket after haplotype codes (XgFBt01‐06). XbF and SmF represent symbiotic fungi isolated from mycangia of *Xylosandrus brevis* and *Scolytoplatypus mikado*, respectively

β‐tubulin sequences of approximately 440 bp had 52.0% G/C content. These fragments varied from 436 to 442 nucleotides. Six haplotypes were defined from 125 isolates. Haplotypes XgFBt01, XgFBt02, XgFBt03, and XgFBt04 were detected only in Type I (Figure 5). Haplotypes XgFBt05 and XgFBt06 were detected only in Types II and III, respectively. The *X. germanus* fungi haplotypes were clustered as a monophyletic group with one strain of *A. hartigii* (EU825654). Type I and II haplotypes were also clustered with high bootstrap value of 65.

**Figure 6:**
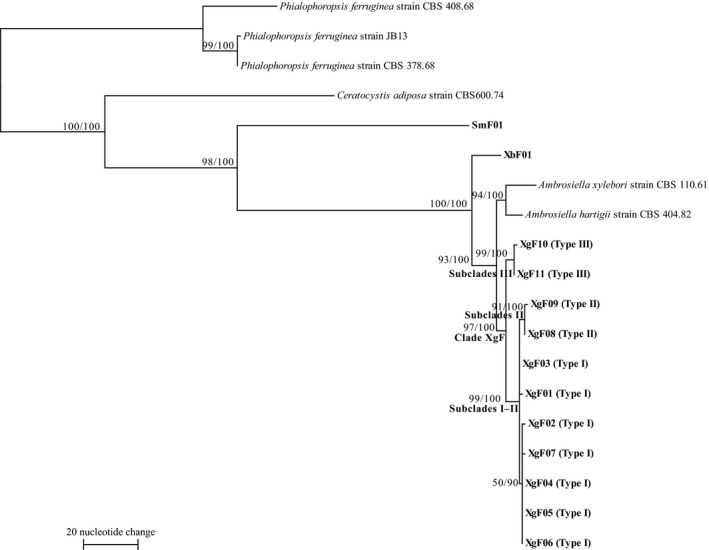
Phylogram obtained from about 2,000 bp of the combined loci (the 18S, the 28S rDNA, and the β‐tubulin gene) of symbiotic fungi isolated from mycangia of *Xylosandrus germanus* and related fungi species. One of 10 maximum‐parsimony (MP) trees. CI = 0.863, RI = 0.913, length = 519 steps. Bootstrap values (left) and branch support values (right) (>50%) are given above the branches. Bold letters indicate sequences obtained in this study. Cultural types (Type I‐III) defined in Figure 1 are shown in bracket after haplotype codes (XgF01‐11). XbF and SmF represent symbiotic fungi isolated from mycangia of *Xylosandrus brevis* and *Scolytoplatypus mikado*, respectively

**Figure 7:**
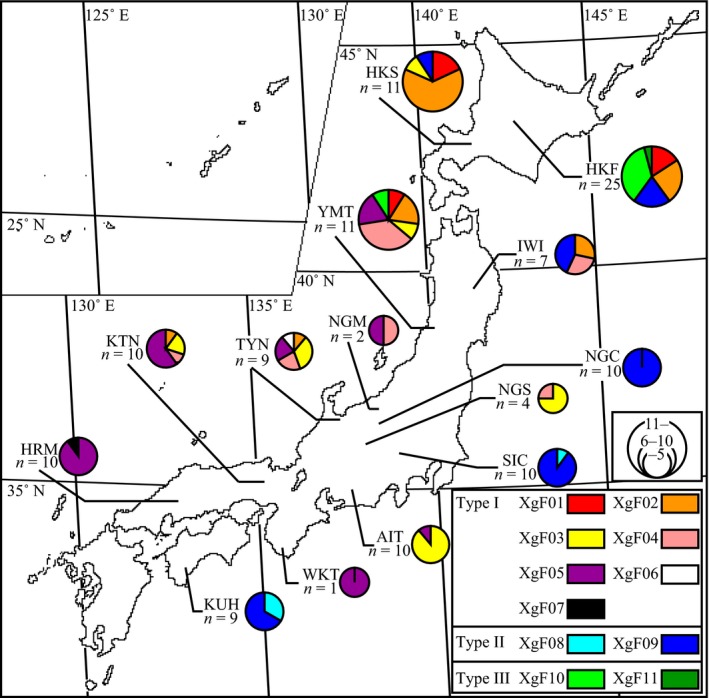
Distribution of haplotypes based on the combined loci (18S rDNA, 28S rDNA, and β‐tubulin genes) of symbiotic fungi across 14 Japanese *Xylosandrus germanus* populations. Pie charts represent the frequencies of the haplotypes in each population. The size of the circles is proportional to the sample size. Population names are defined in Table 1. Cultural types (Types I–III) and haplotype codes (XgF01–11) are defined in Figures 1 and 6, respectively. Type I, II, and III fungi include the XgF01–07, XgF08–09, and XgF10–11 haplotypes, respectively

The ILD test indicated that the 18S, 28S, and β‐tubulin datasets were concordant (*p *=* *.635). On the basis of the three combined loci, 11 multilocus haplotypes were defined from 129 isolates (Table 4). Haplotypes 07 were detected only in Type I (Figure 6). Haplotypes XgF08–09 and XgF10–11 were detected only in Types II and III, respectively. Symbiotic fungi isolated from *X. germanus* mycangia clustered as a monophyletic group with one strain of *A. hartigii*, CBS 404.82, and *A. xylebori*, CBS 110.61. Within this clade, symbiotic fungi of *X. germanus* formed a subclade (clade XgF). Within clade XgF, Type I and II haplotypes formed subclades I–II, and Type III haplotypes were clustered as a monophyletic group. Within subclade I–II, only Type II haplotypes were clustered in a subclade (subclade II) and five Type I haplotypes, XgF02, and XgF04–07, were clustered in a subclade. We compared geographical distribution of seven Type I haplotypes using chi‐square test. Seven Type I haplotypes were not uniformly distributed in Japan (χ^2^‐test, *p *<* *.05; Figure 7). XgF01 was only found in three northern populations (HKF, HKS, and YMT). XgF02 was distributed in four northern populations (HKF, HKS, IWI, and YMT) and in two other populations along the Japan Sea (TYN and KTM). Both XgF03 and XgF04 were found in four northern, central, and western populations (YMT, NGS, TYN, and KTM). Moreover, XgF03 and XgF04 were found in two other populations, HKS and AIT, and IWI and NGM, respectively. XgF05 was detected from seven northern to western populations (YMT, NGM, TYN, AIT, KTM, WKT, and HRM). XgF06 and XgF07 were only found in TYN and HRM, respectively.

**Table 4:** Haplotype frequencies both mitochondrial DNA (*COI*) of *Xylosandrus germanus* and three loci DNA^a^ of symbiotic fungi isolated from its mycangia

Insect clades	Insect haplotypes	Cultural types and haplotypes of fungi											Total
		Type I							Type II		Type III		
		XgF01	XgF02	XgF03	XgF04	XgF05	XgF06	XgF07	XgF08	XgF09	XgF10	XgF11	
A	XgCOI01	6	12	1	—	—	—	—	—	—	—	—	19
	XgCOI02	—	1	—	—	—	—	—	—	—	—	—	1
	XgCOI03	—	3	12	7	19	1	1	—	—	—	—	43
	XgCOI04	—	1	—	—	1	—	—	—	—	—	—	2
	XgCOI05	1	1	—	1	—	—	—	—	—	—	—	3
	XgCOI06	—	—	—	1	—	—	—	—	—	—	—	1
	XgCOI07	—	—	3	—	—	—	—	—	—	—	—	3
	XgCOI08	—	—	—	1	—	—	—	—	—	—	—	1
	XgCOI09	—	—	1	—	—	—	—	—	—	—	—	1
	XgCOI11	—	1	2	1	—	—	—	—	—	—	—	4
	XgCOI12	—	—	—	—	1	—	—	—	—	—	—	1
	XgCOI13	—	—	—	—	1	—	—	—	—	—	—	1
B	XgCOI14	—	—	—	—	—	—	—	—	2	—	—	2
	XgCOI15	—	—	—	—	—	—	—	1	19	—	—	20
	XgCOI16	—	—	—	—	—	—	—	—	7	—	—	7
	XgCOI17	—	—	—	—	—	—	—	2	6	—	—	8
	XgCOI19	—	—	—	—	—	—	—	1	—	—	—	1
C	XgCOI20	—	—	—	—	—	—	—	—	—	10	1	11
	Total	7	19	19	11	22	1	1	4	34	10	1	129

a Combined 18S rDNA, 28S rDNA, and β‐tubulin gene.

**Figure 8:**
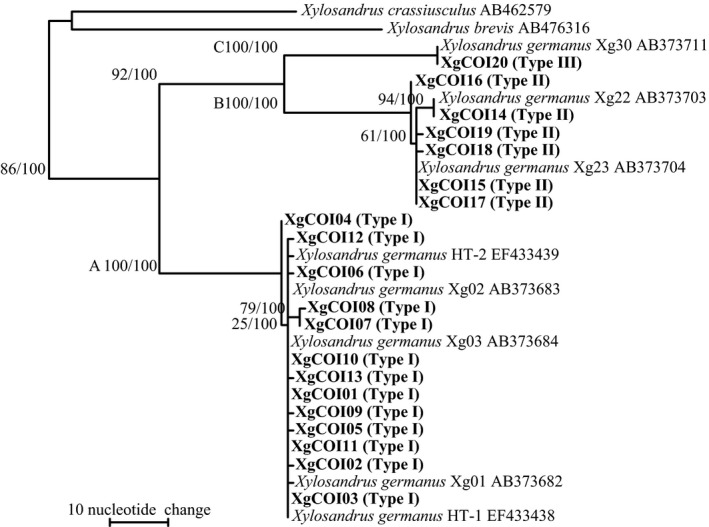
Phylogram obtained from 794 bp of the COI mtDNA from *Xylosandrus germanus*. One of 370 maximum‐parsimony (MP) trees. CI = 0.737, RI = 0.924, length = 223 steps. Bootstrap values (left) and branch support values (right; >50%) are given above the branches. Bold letters indicate sequences obtained in this study. Cultural types (Type I–III) defined in Figure 1 are shown in bracket after haplotype codes (XgCOI01‐20). Capital letters A, B, and C stand for the three clades

Sequencing of *COI* from the mtDNA of *X. germanus* aligned 794 bp. Twenty haplotypes were defined from 133 individuals. These fragments had a 34.6% G/C content. The *X. germanus* haplotypes were clustered as a monophyletic group together with the haplotypes of the outgroups of *X. brevis* (AB476316) and *Xylosandrus crassiusculus* (Motschulsky) (AB462579; Figure 8). The phylogenetic analysis revealed three distinct clades (A–C) with high bootstrap values. Clade A had 13 haplotypes (XgCOI01–13), clade B six haplotypes (XgCOI14–19), and clade C one haplotype (XgCOI20). *X. germanus* clades A, B, and C were unexceptionally associated with symbiotic fungi Types I, II, and III, respectively (Table 4, Appendix 1). However, no specific associations were observed between clade A and Type I at the haplotype level. For example, XgCOI03 of clade A had all haplotypes of Type I fungi in its mycangia, expect for XgF01. The numbers of Type II and III fungi haplotypes were too small to evaluate the relationships between the fungal and beetle haplotypes.

Nucleotide sequences obtained in this study were submitted to the DDBJ/EMBL/GenBank databases (accession numbers: LC140885‐LC140924) (Table 5).

**Table 5:** List of GenBank accession no. of nucleotide sequences obtained in this study

Species	Locus	Haplotype	GenBank accession no.
Fungal symbiont isolated from *X. germanus* mycangium	18S ribosomal RNA	XgF18S01	LC140885
		XgF18S02	LC140886
	28S ribosomal RNA	XgF28S01	LC140889
		XgF28S02	LC140890
		XgF28S03	LC140891
		XgF28S04	LC140892
		XgF28S05	LC140893
	β‐tubulin gene	XgFBt01	LC140897
		XgFBt02	LC140898
		XgFBt03	LC140899
		XgFBt04	LC140900
		XgFBt05	LC140901
		XgFBt06	LC140902
Fungal symbiont isolated from *X. brevis* mycangium	18S ribosomal RNA	XbF18S01	LC140887
	28S ribosomal RNA	XbF28S01	LC140894
	β‐tubulin gene	XbFBt01	LC140903
Fungal symbiont isolated from *S. mikado* mycangium	18S ribosomal RNA	SmF18S01	LC140888
	28S ribosomal RNA	SmF28S01	LC140895
		SmF28S02	LC140896
	β‐tubulin gene	SmFBt01	LC140904
*Xylosandrus germanus*	*COI* mtDNA	XgCOI01	LC140905
		XgCOI02	LC140906
		XgCOI03	LC140907
		XgCOI04	LC140908
		XgCOI05	LC140909
		XgCOI06	LC140910
		XgCOI07	LC140911
		XgCOI08	LC140912
		XgCOI09	LC140913
		XgCOI10	LC140914
		XgCOI11	LC140915
		XgCOI12	LC140916
		XgCOI13	LC140917
		XgCOI14	LC140918
		XgCOI15	LC140919
		XgCOI16	LC140920
		XgCOI17	LC140921
		XgCOI18	LC140922
		XgCOI19	LC140923
		XgCOI20	LC140924

## 4 DISCUSSION

Symbiotic fungi isolated from *X. germanus* mycangia in Japan had all three cultural types (Figures 1 and 2). The three types formed one clade with *A. grosmanniae*,* A. roeperi*,* A. hartigii and A.xylebori in* 18S and 28S (Figures 3 and 4). In β‐tubulin gene, *X. germanus* fungi clustered as a monophyletic group together with *A. beaveri*,* A. hartigii and A. xylebori* clade (Figure 5). In combined three loci, *X. germanus* fungi clustered as a monophyletic group together with *A. hartigii* and *A. xylebori* clade (Figure 6). Therefore, all fungal isolates obtained in this study were identified as closely related species to four species, *A. grosmanniae, A. roeperi, A. hartigii,* and *A. xylebori*. Phylogenetic analyses based on 28S rDNA, β‐tubulin, and the combined three loci revealed that Types I and II haplotypes formed subclade within *X. germanus* fungi clade with high bootstrap values (Figures 4, 5, 6). These results suggest that three types of *X. germanus* fungi, which are distinct from each other as per morphological and phylogenetic characters, are distributed in Japan. These results also suggest that Type III first differentiated from ancestral members, common to all three types, and subsequently, ancestral members of Types I and II have differentiated into Types I and II.

The *COI* haplotypes of *X. germanus* were divided into three distinct lineages (Figure 8). This result was the same that of Ito et al. (2008). The beetles had a specific type of symbiotic fungi for each clade (Figure 8 and Table 4). These results suggest that *X. germanus* are unable to exchange mycangial fungi between clades of beetles. However, horizontal transmission of mycangial fungi may occur within the same beetle lineage, because no specific relationships were found between beetle and fungal haplotypes within same clade (Table 4). Some bark beetles likely exchange their fungi between neighboring nests in the same host tree (Six, 2003; Six & Bentz, 2007). Each Type I haplotype showed a nonrandom distribution on the Japanese archipelago (Figures 7 and 8). These distributions may be formed by *X. germanus*, because fungal dispersion depends on beetle migration. *X. germanus* cannot migrate between Hokkaido and other regions in Japan because of the Tsugaru Strait geographical barrier (Ito et al., 2008). However, scolytine beetles have a flying range of 10–15 km (Gries, 1985; Wood, 1982). Thus, the migration ability of the beetle may regulate fungal dispersion, resulting in the lack of random distribution in the Type I haplotypes. Additionally, *COI* clades of *X. germanus* can be distinguished by the cultural types of its symbiotic fungi, because the clades have strong correlations with the cultural types (Figure 8 and Table 4).

The phylogenetic divergence patterns of the symbiotic fungi did not coincide with those of *X. germanus* (Figures 6 and 8). In mycangial fungi, Type III lineage was sister to a clade containing Type I and Type II lineages (Figure 6). In contrast, ancestral members of *X. germanus* branched into clade A and clades B and C lineages first, and clade B and clade C lineages divided subsequently from the clades B and C (Figure 8). These results suggest that the first fungal differentiation may have occurred together with the first beetle differentiation. In particular, Types I and II and Type III may have concurrently diverged from fungal ancestors when clade A and clades B and C differentiated from beetle ancestors. Ito (2009) showed the differentiation between clade A and a clade composed of clades B and C, and clade B and clade C of *X. germanus* occurred six MYA and 5.2 MYA, respectively. Based on this molecular clock, first differentiations between beetle and their mycangial fungi may have occurred six MYA. *X. germanus* had already developed into three lineages before colonization of the Japanese archipelago (Ito et al., 2008). After colonization, clade A and B beetles secondarily came into contact during the last glacial epoch in Japan (Ito et al., 2008). Type II was not differentiated within subclade I–II (Figure 6), suggesting that clade B ancestors may have symbiotically associated with Type II when clade B occurred and contacted to clade A.

We obtained two important results related to the phylogeny of *X. germanus* and its symbiotic fungi: a single beetle lineage is consistently associated with a single fungal type in the *X. germanus* fungal symbiont system, although more than two types of symbiotic fungi were found in northern populations (Figure 2), and exceptional horizontal transmission in symbiotic fungi between beetles lineages occurred at least once, sustaining novel beetle‐fungus symbiotic relationships. Why are the beetles unable to exchange symbiotic fungi from the existing type to other types? In ambrosia beetles, glandular secretions into the mycangium can facilitate the growth of specific ambrosia fungi (Harrington, 2005; Norris, 1979). Some bark beetles such as the southern pine beetle (*Dendroctonus frontalis* Zimmermann) also have glandular cells in their mycangia and carry one specific fungal symbiont (Bridges, 1985). Thus, it is possible that specific ambrosia fungi lineages in *X. germanus* are selected by mycangia secretion. Colony growth rate on PDA and the competitive race of each fungal type vary according to thermal conditions (Ito & Kajimura, 2011). Some symbiotic fungi of bark beetles also have thermal traits in the field (Six & Bentz, 2007; Six & Paine, 1997; Solheim & Krokene, 1998). The fitness level of ambrosia and bark beetles decreases or increases depending on the symbiotic fungal species used for nutrition (Harrington, 2005; Kajimura, 2000; Six & Bentz, 2007). Therefore, *X. germanus* and their mycangial fungi mutual systems may experience constant selection pressure from environmental and ecological factors. These selection pressures would help maintain the specific relationships between *X. germanus* and their mycangial fungi. Further investigations, particularly those focusing on glandular cells and thermal conditions, will clarify the factors involved in maintaining the *X. germanus*–fungal symbiosis.

## CONFLICT OF INTEREST

None declared.

## AUTHOR CONTRIBUTIONS

M. Ito designed the study, wrote the initial draft of the manuscript, and analyzed and interpreted data in the study. H. Kajimura contributed to interpretation of data, assisted in the preparation of the manuscript, and critically reviewed the manuscript. All authors approved the final version of the manuscript, and agree to be accountable for all aspects of the work in ensuring that questions related to the accuracy or integrity of any part of the work are appropriately investigated and resolved.

## APPENDIX 1 List of cultural types and haplotypes of symbiotic fungi isolated from mycangia of *Xylosandrus germanus* and haplotypes of *X. germanus* samples

### 1.1

**Table:**

Acronym^a^	ID	Cultural types		Fungus haplotypes			Insect haplotypes
			18S	28S	β‐tubulin	Three loci^b^	COI
HKF	HKF‐01	Type‐I	01	01	01	01	01
	HKF‐02	Type‐I	01	01	01	01	01
	HKF‐03	Type‐I	01	02	01	02	01
	HKF‐04	Type‐I	01	02	01	02	01
	HKF‐05	Type‐I	01	02	01	02	01
	HKF‐06	Type‐I	01	01	01	01	01
	HKF‐07	Type‐I	01	01	01	01	01
	HKF‐08	Type‐I	01	02	01	02	02
	HKF‐09	Type‐I	01	02	01	02	01
	HKF‐10	Type‐I	01	02	01	02	01
	HKF‐11	Type‐II	02	01	05	09	14
	HKF‐12	Type‐II	02	01	05	09	14
	HKF‐13	Type‐II	02	01	05	09	15
	HKF‐14	Type‐II	02	01	05	09	15
	HKF‐15	Type‐II	02	01	05	09	15
	HKF‐16	Type‐III	01	04	06	10	20
	HKF‐17	Type‐III	01	04	06	10	20
	HKF‐18	Type‐III	01	04	06	10	20
	HKF‐19	Type‐III	01	04	06	10	20
	HKF‐20	Type‐III	01	04	06	10	20
	HKF‐21	Type‐III	01	04	06	10	20
	HKF‐22	Type‐III	01	04	06	10	20
	HKF‐23	Type‐III	01	05	06	11	20
	HKF‐24	Type‐III	01	04	06	10	20
	HKF‐25	Type‐III	01	04	06	10	20
HKS	HKS‐01	Type‐I	01	02	01	02	01
	HKS‐02	Type‐I	01	01	02	03	01
	HKS‐03	Type‐I	01	01	01	01	01
	HKS‐04	Type‐I	01	01	01	01	01
	HKS‐05	Type‐I	01	02	01	02	01
	HKS‐06	Type‐I	01	02	01	02	01
	HKS‐07	Type‐I	01	02	01	02	01
	HKS‐08	Type‐I	01	02	01	02	01
	HKS‐09	Type‐I	01	02	01	02	01
	HKS‐10	Type‐I	01	02	01	02	01
	HKS‐11	Type‐II	02	01	05	09	15
IWI	IWI‐01	Type‐I	01	02	01	02	03
	IWI‐02	Type‐I	01	02	02	04	03
	IWI‐03	Type‐I	01	02	01	02	03
	IWI‐04	Type‐I	01	02	02	04	03
	IWI‐05	Type‐II	02	01	05	09	15
	IWI‐06	Type‐II	02	01	05	09	15
	IWI‐07	Type‐II	02	01	05	09	15
YMT	YMT‐01	Type‐I	01	02	03	05	03
	YMT‐02	Type‐I	01	01	02	03	03
	YMT‐03	Type‐I	01	02	03	05	04
	YMT‐04	Type‐I	01	02	02	04	03
	YMT‐05	Type‐I	01	01	01	01	05
	YMT‐06	Type‐I	01	02	02	04	03
	YMT‐07	Type‐I	01	02	01	02	05
	YMT‐08	Type‐I	01	02	02	04	05
	YMT‐09	Type‐I	01	02	01	02	04
	YMT‐10	Type‐I	01	02	02	04	03
	YMT‐11	Type‐III	01	04	06	10	20
SIC	SIC‐01	Type‐II	01	01	05	08	15
	SIC‐02	Type‐II	02	01	05	09	15
	SIC‐03	Type‐II	02	01	05	09	15
	SIC‐04	Type‐II	02	01	05	09	15
	SIC‐05	Type‐II	02	01	05	09	15
	SIC‐06	Type‐II	02	01	05	09	15
	SIC‐07	Type‐II	02	01	05	09	15
	SIC‐08	Type‐II	02	01	05	09	15
	SIC‐09	Type‐II	02	01	05	09	15
	SIC‐10	Type‐II	02	01	05	09	15
NGM	NGM‐01	Type‐I	01	02	03	05	03
	NGM‐02	Type‐I	01	02	03	04	06
NGC	NGC‐01	Type‐II	02	01	05	09	15
	NGC‐02	Type‐II	02	01	05	09	16
	NGC‐03	Type‐II	02	01	05	09	16
	NGC‐04	Type‐II	02	01	05	09	16
	NGC‐05	Type‐II	02	01	05	09	15
	NGC‐06	Type‐II	02	01	05	09	16
	NGC‐07	Type‐II	02	01	05	09	16
	NGC‐08	Type‐II	02	01	05	09	15
	NGC‐09	Type‐II	02	01	05	09	16
	NGC‐10	Type‐II	02	01	05	09	16
NGS	NGS‐01	Type‐I	01	01	02	03	07
	NGS‐02	Type‐I	01	02	02	03	07
	NGS‐03	Type‐I	01	01	02	04	08
	NGS‐04	Type‐I	01	01	02	03	07
TYN	TYN‐01	Type‐I	01	01	02	03	03
	TYN‐02	Type‐I	01	02	02	04	03
	TYN‐03	Type‐I	01	02	—	—	10
	TYN‐04	Type‐I	01	02	02	04	11
	TYN‐05	Type‐I	01	01	02	03	11
	TYN‐06	Type‐I	01	02	03	05	03
	TYN‐07	Type‐I	01	02	01	02	03
	TYN‐08	Type‐I	01	01	02	03	11
	TYN‐09	Type‐I	01	02	03	05	03
	TYN‐10	Type‐I	01	02	04	06	03
AIT	AIT‐01	Type‐I	01	02	03	05	03
	AIT‐02	Type‐I	01	01	02	03	03
	AIT‐03	Type‐I	01	01	02	03	03
	AIT‐04	Type‐I	01	01	02	03	03
	AIT‐05	Type‐I	01	01	02	03	03
	AIT‐06	Type‐I	01	01	02	03	09
	AIT‐07	Type‐I	01	01	02	03	03
	AIT‐08	Type‐I	01	01	02	03	03
	AIT‐09	Type‐I	01	01	02	03	03
	AIT‐10	Type‐I	01	01	02	03	03
KTN	KTN‐01	Type‐I	01	02	03	05	03
	KTN‐02	Type‐I	01	02	03	05	03
	KTN‐03	Type‐I	01	02	03	05	03
	KTN‐04	Type‐I	01	02	01	02	11
	KTN‐05	Type‐I	01	02	03	05	03
	KTN‐06	Type‐I	01	01	02	03	03
	KTN‐07	Type‐I	01	02	03	05	12
	KTN‐08	Type‐I	01	01	02	03	03
	KTN‐09	Type‐I	01	02	03	05	03
	KTN‐10	Type‐I	01	02	02	04	03
WKT	WKT‐01	Type‐I	01	02	03	05	03
HRM	HRM‐01	Type‐I	01	03	03	07	03
	HRM‐02	Type‐I	01	02	—	—	03
	HRM‐03	Type‐I	01	02	03	05	03
	HRM‐04	Type‐I	01	02	03	05	03
	HRM‐05	Type‐I	01	02	03	05	03
	HRM‐06	Type‐I	01	02	03	05	13
	HRM‐07	Type‐I	01	02	03	05	03
	HRM‐08	Type‐I	01	02	03	05	03
	HRM‐09	Type‐I	01	02	03	05	03
	HRM‐10	Type‐I	01	02	03	05	03
	HRM‐11	Type‐I	01	02	03	05	03
KUH	KUH‐01	Type‐II	02	01	05	09	17
	KUH‐02	Type‐II	02	01	05	09	17
	KUH‐03	Type‐II	02	01	05	09	17
	KUH‐04	Type‐II	02	01	—	—	18
	KUH‐05	Type‐II	02	01	05	09	17
	KUH‐06	Type‐II	02	01	05	09	17
	KUH‐07	Type‐II	01	01	05	08	19
	KUH‐08	Type‐II	02	01	05	09	17
	KUH‐09	Type‐II	01	01	05	08	17
	KUH‐10	Type‐II	01	01	05	08	17

a Site names are defined in Table 1.

b Conbined 18S rDNA, 28S rDNA and β‐tubulin gene.
